# Association of metformin exposure with low risks of frailty and adverse outcomes in patients with diabetes

**DOI:** 10.1186/s40001-023-01017-6

**Published:** 2023-02-03

**Authors:** Pan Liu, Yiming Pan, Yu Song, Yaru Zhou, Wanshu Zhang, Xiaojun Li, Jiatong Li, Yun Li, Lina Ma

**Affiliations:** Department of Geriatrics, National Clinical Research Center for Geriatric Diseases, Xuanwu Hospital Capital Medical University, Beijing, China

**Keywords:** Metformin, Frailty, Diabetes, Aging, Adverse outcomes

## Abstract

**Background:**

Diabetes is an independent risk factor of frailty, which increases adverse outcomes in patients with diabetes. Metformin is a common antidiabetic drug in clinical practice. Insulin resistance and chronic inflammation are the two common mechanisms of diabetes and frailty, as well as the main targets of metformin. Research suggested that metformin has anti-aging potential. However, few studies focus on the relationship between metformin and frailty. Thus, we aimed to explore whether metformin was associated with a low risk of frailty and other adverse outcomes in diabetic patients.

**Methods:**

A total of 422 patients (≥ 40 years old) with type 2 diabetes were recruited. Frailty was defined by the Fried phenotype. General information and metformin exposure data were collected, and comprehensive geriatric assessment and laboratory tests were performed. Follow-up was conducted after 4.5 years. The primary outcome was the combined endpoint of cardiovascular events, cerebrovascular events, readmission, and death. Binary logistic regression analysis was used to analyze the association of metformin with frailty. Survival analysis was performed using Cox proportional hazards models.

**Results:**

The total prevalence of frailty was 19.4% among the participants with diabetes. 13.1% of patients in the metformin group and 28.2% in the non-metformin group had frailty. Metformin was inversely associated with frailty after adjusting for age, sex, duration, blood glucose levels, target organ damage, comorbidities, and polypharmacy. Further longitudinal analysis showed that metformin was also independently associated with a low risk of combined primary outcomes after adjusting for multiple covariables, while frailty was related to an increased risk of the combined primary outcomes. In the non-frail group, metformin was associated with a decreased risk of combined primary outcomes after adjustment for age and sex. However, the protective effect of metformin on adverse outcomes was not found in frail participants with diabetes.

**Conclusions:**

Metformin use is associated with a reduced risk of frailty. In addition, frailty may attenuate the protective effects of metformin on adverse outcomes in diabetic patients. The early identification and prevention of frailty progression may help enhance the benefits of metformin in patients with diabetes.

**Supplementary Information:**

The online version contains supplementary material available at 10.1186/s40001-023-01017-6.

## Background

Diabetes is an age-related chronic metabolic disease. In 2021, 537 million (10.5%) adults aged 20–79 years were estimated to live with diabetes. The number is predicted to rise to 643 million by 2030 and 783 million by 2045 [1]. The overall prevalence of diabetes diagnosed by World Health Organization criteria in Chinese adults was 11.2% in 2017, which was increased by 1.5% over the past 10 years [2]. Diabetes is associated with many complications, such as macrovascular and microvascular target organ damage, leading to serious public health problems. Frailty is a clinical state with multiple decreased functions and more vulnerability to stress, resulting in a high risk of adverse outcomes, such as falls, disability, hospitalization, dependency, and even death [3, 4]. Diabetes, a risk factor for frailty, can increase the prevalence of frailty [3, 5]. Meanwhile, frailty also worsens health conditions in older adults with diabetes [6]. Research has found that patients with diabetes are more likely to exhibit reduced leg muscle mass with function loss [7, 8], which might be associated with frailty performance. Chronic inflammation and insulin resistance are two of the most common pathological mechanisms of diabetes and frailty [8].

Metformin is a strongly recommended first-line drug for the treatment of diabetes as well as a potential anti-aging medicine. Evidence from both clinical and pre-clinical studies supports the benefits of metformin in improving healthspan and expanding lifespan [9]. Moreover, metformin can reduce the risk of all-cause mortality and age-related diseases, such as cardiovascular disease and cancer in non-diabetic adults [10]. These anti-aging effects are mainly from non-diabetes research, which is associated with improvement in deregulated nutrient sensing, altered intercellular communication, proteostasis disorders, and genomic instability [11]. In addition, metformin inhibits the hallmarks of aging such as mitochondrial dysfunction, cellular senescence, and stem cell exhaustion [11].

Diabetes and frailty often co-exist in adults. Only a few studies have investigated the relationship between frailty and metformin use, and have found that metformin was associated with a low risk of frailty evaluated by the Frailty Index-40 and frailty-related diseases in older male veterans with diabetes [12, 13]. However, the protective effects of metformin on frailty-related skeletal muscle remain controversial [14–16]. Although previous studies have shown that metformin improves multiple aging phenotypes, whether it has a similar effect on age-related frailty requires further investigation. Our objective was mainly to explore the association between metformin use and frailty in patients with diabetes.

## Methods

### Study design and participants

Data were obtained from the ongoing cohort of the Physiological Model for Frailty and Resilience Study (SMART). The inclusion criteria were a clinical diagnosis of type 2 diabetes and patients aged 40 years or older. Patients with type 1 diabetes or severe multiple organ dysfunction were excluded. The clinical data for the first participant were collected in January 2018. Ultimately, 422 participants met the inclusion criteria. The median follow-up time was 21.0 months (95% CI 19.3–22.7) for participants in the study. All participants voluntarily participated in this study and signed an informed consent form. The study design was approved by the Ethics Review Board of Xuanwu Hospital Capital Medical University. Information was only collected after written consent was obtained from all participants. This study was conducted in accordance with the Declaration of Helsinki.

### Metformin exposure

Individuals treated with metformin were considered exposed, including monotherapy and a combination of other antidiabetic therapy. Those using sulfonylureas, glinides, glucosidase inhibitors, thiazolidinediones, dipeptidyl peptidase 4 (DPP-4) inhibitors, sodium–glucose transport protein 2 (SGLT2) inhibitors, glucagon-like peptide-1 (GLP-1) agonist, insulin, and other antidiabetics, or lifestyle management (exercise and/or diet control) were considered as not exposed.

### Measurements

General characteristics of the participants, including age and sex, duration of diabetes, comorbidities, and/or target organ damage, were obtained from electronic medical records. Weight, height, waist, and hip were measured on admission, and the body mass index (BMI) (kg/m^2^) and waist-to-hip ratio were calculated.

### Frailty assessment

Frailty was assessed by Fried phenotype, which consists of five items [3]. Weakness was evaluated by grip strength with a lowest 20% of the Chinese older population adjusted by sex and BMI [17]. It was measured with a handgrip dynamometer (CAMRY, EH101) in this study. Slowing walking speed was evaluated by the 4 m walking test with a lowest 20% of the Chinese older adults adjusted by sex and height [18]. Exhaustion was identified by self-report using two questions from the Centre of Epidemiology Studies Depression Scale [19]. Inactivity was defined as little or no activity, or less than 3 h of outdoor activity per week. Weight loss was measured by unintentionally losing  ≥ 4.5 kg or 5.0% in the past year or BMI  < 18.5 kg/m^2^. Frailty was defined as the presence of three or more of the above phenotypes, and non-frailty was that met 0 to 2 items.

### Quality of life assessment

Medical Outcomes Study’s 36-Item Short Form Health Survey (SF-36) is a widely used method to evaluate the quality of life and is also suitable for Chinese adults [20]. It includes eight dimensions of physical functioning, role physical, body pain, general health, vitality, social functioning, role-emotional, and mental health. Health transition represents the overall health changes in the past year. The SF-36 total scores are the sum of each dimension [21, 22]. The higher the SF-36 score, the worse quality of life.

### Laboratory tests

Fasting blood and morning urine were collected. The levels of fasting plasma glucose, hemoglobin A1C, triglyceride, total cholesterol, high-density lipoprotein, low-density lipoprotein, creatinine, blood urea nitrogen, prealbumin, albumin, hemoglobin, C-reactive protein (CRP), D-dimer, fibrinogen, and N-terminal pro-B-type natriuretic peptide (NT-proBNP) in serum were assayed. Urine microalbuminuria/creatinine (mALB/Cre) was calculated.

### Outcomes

The primary outcome was the combined endpoint of cardiovascular events, cerebrovascular events, readmission, and death. The cardiovascular events were assessed by Major Adverse Cardiovascular Events (MACE), which include unstable angina, acute myocardial infarction, severe arrhythmia, heart failure, and coronary heart disease death in this study. The cerebrovascular events included acute stroke and stroke death. For those patients already with a history of coronary artery disease and stroke at baseline, new-onset acute cardiovascular and cerebrovascular events during the follow-up period are used as the criterion. Readmission was defined as hospitalization within 30 days and hospitalization due to acute cardiovascular and cerebrovascular events during follow-up. All information was collected from patients and medical system records.

### Statistical analysis

Continuous variables were described with a mean (± standard deviation) or median (quartile) and analyzed by Student’s t-test and Mann–Whitney U test. Frequency (%) and Chi-square test were used to describe and analyze the categorical variables, respectively. Binary logistic regression was used to analyze the independent effects of metformin on frailty and reported the odds ratios (OR) and 95% confidence intervals (CIs). Furthermore, survival analysis was estimated by the Kaplan–Meier method and the log-rank test was used to evaluate the difference between the metformin and non-metformin groups. Cox proportional-hazards models were used to assess the effects of metformin on the survival of patients with diabetes by adjusting age, sex, and frailty. Baseline variables that were considered clinically relevant or that showed a univariate relationship with outcome were entered into logistic regression analysis and multivariate Cox proportional-hazards regression model. Data were analyzed using IBM SPSS version 26.0 software (Inc., Chicago, IL, USA) and GraphPad Prism 7.0 software (GraphPad Software Inc., CA, USA). *P*-value < 0.05 is defined as a statistically significant difference.

## Results

### General characteristics in non-frail and frail participants with diabetes

The mean age was 70.35 (± 10.71) years, and 64.0% (*n* = 270) were men. The prevalence of frailty was 19.4% (*n* = 82) in participants with diabetes. The general characteristics of the non-frail and frail groups are compared in Table 1. Compared with non-frail patients, frail patients were older and had a higher prevalence of hypertension, coronary atherosclerotic heart disease, chronic kidney disease, stroke, osteoarthritis, cancer, peripheral vascular disease, diabetic peripheral neuropathy, and polypharmacy. Frail patients had lower grip strength, slower walking speed, and worse SF-36 scores than that non-frail. A total of 245 patients with diabetes were treated with metformin, with 32 (39.0%) in the frail group and 213 (62.6%) in the non-frail group (Table 1). However, there was no difference in other antidiabetic treatments and duration of diabetes between the two groups.

**Table 1:** Characteristics of non-frail and frail participants with diabetes

Variable	Non-frail (*n* = 340)	Frail (*n* = 82)	*P* value
Age (years)	67.84 ± 9.60	80.76 ± 8.65	< 0.001
Female (n, %)	124 (36.5)	28 (34.1)	0.694
Smoking (n, %)	158 (46.5)	36 (43.9)	0.675
Drinking (n, %)	130 (38.3)	23 (28.4)	0.094
WHR	0.95 ± 0.09	1.08 ± 1.03	0.884
BMI (kg/m^2^)	25.85 ± 3.19	25.25 ± 4.10	0.226
Duration (years)	11.18 ± 8.62	12.43 ± 8.67	0.265
Antidiabetic treatments			
Lifestyle management (n, %)	42 (12.4)	14 (17.1)	0.258
Metformin (n, %)	213 (62.6)	32 (39.0)	< 0.001
Sulfonylureas (n, %)	60 (17.6)	14 (17.1)	0.902
Glinide (n, %)	17 (5.0)	6 (7.3)	0.576
Glucosidase inhibitor (n, %)	140 (41.2)	40 (48.8)	0.211
Thiazolidinedione (n, %)	21 (6.2)	8 (5.6)	0.250
DPP-4 inhibitors (n, %)	27 (7.9)	3 (3.7)	0.176
SGLT2 inhibitors (n, %)	19 (5.6)	4 (4.9)	0.799
GLP-1 agonist (n, %)	7 (2.1)	0 (0.0)	0.407
Insulin (n, %)	100 (29.4)	21 (25.6)	0.494
Other medicines (n, %)	3 (0.9)	1 (1.2)	0.580
Comorbidity			
Hypertension (n, %)	256 (75.3)	74 (90.2)	0.003
CAD (n, %)	111 (32.6)	50 (61.0)	< 0.001
COPD (n, %)	13 (3.8)	5 (6.1)	0.542
CKD (n, %)	34 (10.0)	22 (26.8)	< 0.001
Stoke (n, %)	58 (17.1)	30 (36.6)	< 0.001
Osteoarthritis (n, %)	52 (15.3)	29 (35.4)	< 0.001
Cancer (n, %)	32 (9.4)	23 (28.0)	< 0.001
Target organ damage			
PVD (n, %)	43 (12.6)	23 (28.0)	0.001
DR (n, %)	45 (13.2)	7 (8.5)	0.245
DPN (n, %)	44 (12.9)	19 (23.2)	0.020
Polypharmacy (n, %)	203 (59.7)	68 (82.9)	< 0.001
Grip strength (kg)	30.44 ± 9.52	18.24 ± 7.07	< 0.001
Walking speed (m/s)	1.01 ± 0.26	0.59 ± 0.22	< 0.001
SF-36 score	100.65 ± 11.36	115.49 ± 8.53	< 0.001

Data for continuous variables are presented as mean ± (standard deviation) or median (interquartile range). Data for categorical variables are presented as n (percentage)

WHR, waist-to-hip ratio; BMI, body mass index; DPP-4, dipeptidyl peptidase 4; SGLT2, sodium–glucose transport protein 2; GLP-1, glucagon-like peptide-1; CAD, coronary atherosclerotic heart disease; COPD, chronic obstructive pulmonary disease; CKD, chronic kidney disease; PVD, peripheral vascular disease; DR, diabetic retinopathy; DPN, diabetic peripheral neuropathy

Table 2 exhibits the laboratory test results of the non-frail and frail groups. Compared with non-frail participants, frail patients had higher levels of creatinine, blood urea nitrogen, urine mALB/Cre and lower levels of albumin and hemoglobin. Higher D-dimer, fibrinogen, and NT-proBNP levels were observed in diabetic patients with frailty. However, no difference was found between the two groups regarding blood glucose and lipid biomarkers. In accordance with these variables, univariate binary logistic regression analysis of risk factors for frailty showed similar findings (Additional file 1: Table S1).

**Table 2:** Laboratory tests of non-frail and frail patients with diabetes

Variable	Non-frail (*n* = 340)	Frail (*n* = 82)	*P* value
FPG (mmol/L)	7.18 ± 2.36	7.16 ± 3.27	0.079
HbA1c (%)	7.37 ± 1.52	7.06 ± 1.33	0.067
Triglyceride (mmol/L)	1.79 ± 0.79	1.47 ± 0.70	0.379
Total cholesterol (mmol/L)	4.25 ± 1.15	4.04 ± 1.21	0.044
HDL (mmol/L)	1.12 ± 0.32	1.07 ± 0.30	0.214
LDL (mmol/L)	2.48 ± 0.90	2.39 ± 1.00	0.242
Creatinine (umol/L)	70.80 ± 40.76	94.89 ± 64.03	< 0.001
BUN (mmol/L)	6.19 ± 2.19	8.92 ± 4.96	< 0.001
Prealbumin (mg/L)	248.31 ± 121.64	222.26 ± 65.25	0.062
Albumin (mg/L)	40.14 ± 3.88	37.29 ± 5.69	< 0.001
Hemoglobin (g/L)	135.98 ± 17.41	124.27 ± 21.90	< 0.001
CRP (mg/L)	2.13 (1.90, 4.00)	3.00 (2.00, 5.00)	0.222
D-Dimer (ug/mL)	0.29 (0.20, 0.45)	0.59 (0.37, 1.00)	< 0.001
Fibrinogen (g/L)	3.38 ± 0.72	3.66 ± 0.98	0.016
NT-proBNP (pg/mL)	74.85 (39.75, 197.25)	348.5 (103.98, 781.75)	< 0.001
Urine mALB/Cre	1.50 (0.30, 7.30)	7.00 (0.91, 28.50)	< 0.001

Data for continuous variables are presented as mean ± (standard deviation) or median (interquartile range)

FPG, fasting plasma glucose; HbA1c, hemoglobin A1c; HDL, high-density lipoprotein; LDL, low-density lipoprotein; BUN, blood urea nitrogen; CRP, C-reactive protein; NT-proBNP, N-terminal pro-B-type natriuretic peptide; mALB/Cre, microalbuminuria/creatinine

### Metformin exposure in different frailty groups

Compared with the non-metformin group, the metformin group was younger and had a higher BMI, a higher risk of coronary atherosclerotic heart disease, chronic kidney disease, osteoarthritis, and peripheral vascular disease, a lower proportion of frailty, greater grip strength, and faster walking speed (Table 3). However, these differences in physical performance did not exist in the subgroup analysis of the non-frail and frail participants (Table 4). Both frail and non-frail patients treated with metformin had a lower prevalence of coronary atherosclerotic heart disease and chronic kidney disease than those not treated with metformin (Tables 3 and 4). Non-frail participants taking metformin had a lower prevalence of osteoarthritis and peripheral vascular disease and higher levels of fasting plasma glucose, hemoglobin A1c, triglyceride, prealbumin, albumin, and hemoglobin than those not taking metformin (Tables 4 and 5), which was in accordance with the results in Table 6. However, these differences did not exist in the frail group. Frail patients treated with metformin had lower levels of CRP than that with non-metformin (Table 5). Participants taking metformin were more likely to have lower D-dimer and NT-proBNP levels (Table 5) and better quality of life assessed by the SF-36 score (Table 4), consistent with the findings in Table 6 and 3, respectively.

**Table 3:** Characteristics of non-metformin and metformin groups

Variable	Non-metformin (*n* = 177)	Metformin (*n* = 245)	*P* value
Age (years)	74.80 ± 11.01	67.14 ± 9.27	< 0.001
Female (n, %)	62 (35.0)	90 (36.7)	0.719
Smoking (n, %)	76 (42.9)	118 (48.2)	0.288
Drinking (n, %)	53 (29.9)	100 (41.2)	0.018
WHR	1.00 ± 0.65	0.95 ± 0.10	0.305
BMI (kg/m^2^)	25.07 ± 3.64	26.21 ± 3.10	0.001
Duration (years)	11.12 ± 9.20	11.65 ± 8.20	0.538
Antidiabetic treatments			
Sulfonylureas (n, %)	29 (16.4)	45 (18.4)	0.597
Glinide (n, %)	11 (6.2)	12 (4.9)	0.557
Glucosidase inhibitor (n, %)	78 (44.1)	102 (41.6)	0.618
Thiazolidinedione (n, %)	7 (4.0)	22 (9.0)	0.044
DPP-4 inhibitors (n, %)	9 (5.1)	21 (8.6)	0.169
SGLT2 inhibitors (n, %)	3 (1.7)	20 (8.2)	0.004
GLP-1 agonist (n, %)	2 (1.1)	5 (2.0)	0.704
Insulin (n, %)	51 (28.8)	70 (28.6)	0.957
Other medicines (n, %)	1 (0.6)	3 (1.2)	0.643
Comorbidity			
Hypertension (n, %)	145 (81.9)	185 (75.5)	0.116
CAD (n, %)	88 (49.7)	73 (29.8)	< 0.001
COPD (n, %)	11 (6.2)	7 (2.9)	0.092
CKD (n, %)	40 (22.6)	16 (6.5)	< 0.001
Stoke (n, %)	39 (22.0)	49 (20.0)	0.612
Osteoarthritis (n, %)	45 (25.4)	36 (14.7)	0.006
Cancer (n, %)	27 (15.3)	28 (11.4)	0.249
Target organ damage			
PVD (n, %)	38 (21.5)	28 (11.4)	0.005
DR (n, %)	17 (9.6)	35 (14.3)	0.149
DPN (n, %)	22 (12.4)	41 (16.7)	0.221
Polypharmacy (n, %)	118 (66.7)	153 (62.4)	0.372
Frailty (n, %)	50 (28.2)	32 (13.1)	< 0.001
Grip strength (kg)	26.70 ± 10.43	29.30 ± 10.02	0.014
Walking speed (m/s)	0.91 ± 0.30	0.98 ± 0.29	0.038
Exhaustion (n, %)	77 (46.7)	81 (34.9)	0.018
Inactivity (n, %)	56 (35.2)	42 (19.7)	< 0.001
Weight Loss (n, %)	25 (14.8)	30 (12.7)	0.547
SF-36 score	109.22 ± 12.02	115.16 ± 9.11	< 0.001

Data for continuous variables are presented as mean ± (standard deviation) or median (interquartile range). Data for categorical variables are presented as n (percentage)

WHR, waist-to-hip ratio; BMI, body mass index; DPP-4, dipeptidyl peptidase 4; SGLT2, sodium–glucose transport protein 2; GLP-1, glucagon-like peptide-1; CAD, coronary atherosclerotic heart disease; COPD, chronic obstructive pulmonary disease; CKD, chronic kidney disease; PVD, peripheral vascular disease; DR, diabetic retinopathy; DPN, diabetic peripheral neuropathy

**Table 4:** Subgroup analysis of characteristics of non-frail and frail patients with diabetes

Variable	Non-frail (*n* = 340)			Frail (*n* = 82)		
	Non-metformin (*n* = 127)	Metformin (*n* = 213)	*P* value	Non-metformin (*n* = 50)	Metformin (*n* = 32)	*P* value
Age (years)	71.2 ± 10.39	65.84 ± 8.51	< 0.001	83.95 ± 6.22	75.78 ± 9.62	< 0.001
Female (n, %)	44 (34.6)	80 (37.6)	0.589	18 (36.0)	10 (31.3)	0.658
WHR	0.95 ± 0.07	0.95 ± 0.10	0.220	1.14 ± 1.28	0.97 ± 0.06	0.211
BMI (kg/m^2^)	25.07 ± 3.44	26.32 ± 2.94	0.001	25.05 ± 4.17	25.55 ± 4.03	0.582
Smoking (n, %)	55 (43.3)	103 (48.4)	0.366	21 (42.0)	15 (46.9)	0.664
Drinking (n, %)	45 (35.4)	85 (40.1)	0.393	8 (16.0)	15 (48.4)	0.002
Duration (years)	10.44 ± 9.34	11.63 ± 8.12	0.048	12.85 ± 8.60	11.78 ± 8.87	0.415
Comorbidity						
Hypertension (n, %)	100 (78.7)	156 (73.2)	0.255	45 (90.0)	29 (90.6)	0.926
CAD (n, %)	53 (41.7)	58 (27.2)	0.006	35 (70.0)	15 (46.9)	0.036
COPD (n, %)	6 (4.7)	7 (3.3)	0.706	5 (100.0)	0 (0.0)	0.078
CKD (n, %)	20 (15.7)	14 (6.6)	0.006	20 (40.0)	2 (6.3)	0.001
Stoke (n, %)	24 (18.9)	34 (16.0)	0.486	15 (30.0)	15 (46.9)	0.122
Osteoarthritis (n, %)	26 (20.5)	26 (12.2)	0.041	19 (38.0)	10 (31.3)	0.533
Cancer (n, %)	10 (7.9)	22 (10.3)	0.453	17 (34.0)	6 (18.8)	0.134
Target organ damage						
PVD (n, %)	22 (17.3)	21 (9.9)	0.045	16 (32.0)	7 (21.9)	0.319
DR (n, %)	13 (10.2)	32 (15.0)	0.208	4 (8.0)	3 (9.4)	0.564
DPN (n, %)	13 (10.2)	31 (14.6)	0.251	9 (18.0)	10 (31.3)	0.165
Polypharmacy (n, %)	77 (60.6)	126 (59.2)	0.789	41 (82.0)	27 (84.4)	0.780
Grip strength (kg)	29.86 ± 9.53	30.78 ± 9.52	0.383	17.62 ± 7.08	19.09 ± 7.10	0.304
Walking speed (m/s)	0.99 ± 0.26	1.02 ± 0.27	0.361	0.55 ± 0.19	0.64 ± 0.24	0.109
SF-36 score	113.32 ± 10.14	116.76 ± 7.16	0.008	98.10 ± 9.46	104.34 ± 12.94	0.026

Data for continuous variables are presented as mean ± (standard deviation) or median (interquartile range). Data for categorical variables are presented as n (percentage)

WHR, waist-to-hip ratio; BMI, body mass index; CAD, coronary atherosclerotic heart disease; COPD, chronic obstructive pulmonary disease; CKD, chronic kidney disease; PVD, peripheral vascular disease; DR, diabetic retinopathy; DPN, diabetic peripheral neuropathy

**Table 5:** Subgroup analysis of laboratory tests of frail and non-frail patients with diabetes

Variable	Non-frail (*n* = 340)			Frail (*n* = 82)		
	Non-metformin (*n* = 127)	Metformin (*n* = 213)	*P* value	Non-metformin (*n* = 50)	Metformin (*n* = 32)	*P* value
FPG (mmol/L)	6.44 ± 2.02	7.63 ± 2.44	< 0.001	7.46 ± 3.58	6.69 ± 2.70	0.497
HbA1c (%)	6.97 ± 1.32	7.61 ± 1.58	< 0.001	6.82 ± 0.96	7.45 ± 1.70	0.244
Triglyceride (mmol/L)	1.52 ± 1.03	1.96 ± 2.11	0.009	1.42 ± 0.63	1.56 ± 0.80	0.509
Total cholesterol (mmol/L)	4.20 ± 1.16	4.28 ± 1.15	0.478	4.05 ± 0.17	4.02 ± 1.31	0.686
HDL (mmol/L)	1.18 ± 0.38	1.09 ± 0.26	0.060	1.05 ± 0.31	1.10 ± 0.27	0.354
LDL (mmol/L)	2.48 ± 0.95	2.48 ± 0.87	0.747	2.39 ± 0.94	2.39 ± 1.11	0.853
Creatinine (umol/L)	78.43 ± 61.16	66.29 ± 19.43	0.084	102.46 ± 74.29	83.06 ± 41.84	0.172
BUN (mmol/L)	6.46 ± 2.70	6.04 ± 1.80	0.120	9.84 ± 5.86	7.49 ± 2.56	0.101
Prealbumin (mg/L)	228.33 ± 49.16	260.22 ± 147.79	< 0.001	219.1 ± 74.34	227.19 ± 48.47	0.704
Albumin (mg/L)	39.67 ± 4.15	40.41 ± 3.68	0.033	36.94 ± 5.96	37.84 ± 5.28	0.456
Hemoglobin (g/L)	134.06 ± 22.03	137.13 ± 13.90	0.016	123.16 ± 18.36	126.00 ± 26.74	0.287
CRP (mg/L)	2.00 (1.94, 4.00)	2.19 (1.85, 4.00)	0.822	3.47 (2.00, 5.19)	2.00 (1.64, 3.07)	0.017
D-Dimer (ug/mL)	0.33 (0.25, 0.54)	0.26 (0.19, 040)	< 0.001	0.73 (0.48, 1.53)	0.41 (0.32, 0.70)	0.002
Fibrinogen (g/L)	3.41 ± 0.74	3.36 ± 0.71	0.620	3.71 ± 0.71	3.60 ± 0.84	0.945
NT-proBNP (pg/mL)	128.00 (50.03, 241.50)	57.90 (35.98, 127.25)	< 0.001	493.50 (221.25, 1367.50)	178.00 (58.60, 394.50)	0.004
Urine mALB/Cre	1.43 (0.35, 8.36)	1.60 (0.30, 7.30)	0.770	3.80 (0.68, 52.80)	17.40 (1.60, 27.10)	0.723

Data for continuous variables are presented as mean ± (standard deviation) or median (interquartile range)

FPG, fasting plasma glucose; HbA1c, hemoglobin A1c; HDL, high-density lipoprotein; LDL, low-density lipoprotein; BUN, blood urea nitrogen; CRP, C-reactive protein; NT-proBNP, N-terminal pro-B-type natriuretic peptide; mALB/Cre, microalbuminuria/creatinine

**Table 6:** Laboratory tests of non-metformin and metformin groups

Variable	Non-metformin (*n* = 177)	Metformin (*n* = 245)	*P* value
FPG (mmol/L)	6.73 ± 2.59	7.50 ± 2.49	0.002
HbA1c (%)	6.92 ± 1.23	7.59 ± 1.59	< 0.001
Triglyceride (mmol/L)	1.49 ± 0.93	1.90 ± 1.99	0.005
Total cholesterol (mmol/L)	4.16 ± 1.16	4.25 ± 1.17	0.442
HDL (mmol/L)	1.14 ± 0.37	1.09 ± 0.26	0.086
LDL (mmol/L)	2.45 ± 0.94	2.47 ± 0.90	0.819
Creatinine (umol/L)	85.22 ± 65.82	68.45 ± 24.14	0.001
BUN (mmol/L)	7.41 ± 4.14	6.23 ± 1.98	< 0.001
Prealbumin (mg/L)	225.72 ± 57.32	255.91 ± 139.28	0.007
Albumin (mg/L)	38.90 ± 4.87	40.08 ± 4.01	0.007
Hemoglobin (g/L)	130.98 ± 21.57	135.67 ± 16.51	0.016
CRP (mg/L)	2.34 (2.00, 4.58)	2.05 (1.84, 4.00)	0.823
D-Dimer (ug/mL)	0.41 (0.26, 0.70)	0.28 (0.19, 0.43)	0.001
Fibrinogen (g/L)	3.50 ± 0.85	3.39 ± 0.73	0.158
NT-proBNP (pg/mL)	188.00 (73.5, 495.25)	67.05 (37.00, 181.50)	0.007
Urine mALB/Cre	1.64 (0.44, 14.80)	1.85 (0.30, 10.03)	0.021

Data for continuous variables are presented as mean ± (standard deviation) or median (interquartile range)

FPG, fasting plasma glucose; HbA1c, hemoglobin A1c; HDL, high-density lipoprotein; LDL, low-density lipoprotein; BUN, blood urea nitrogen; CRP, C-reactive protein; NT-proBNP, N-terminal pro-B-type natriuretic peptide; mALB/Cre, microalbuminuria/creatinine

### Association between metformin and frailty

Frailty was considered the dependent variable in the study, and regression models were constructed to explore the relationship between metformin and frailty. Univariate regression analysis found that metformin was negatively associated with frailty (B = − 0.963, OR = 0.382; 95%CI 0.233–0.626) (Additional file 1: Table S1). After adjusting for age, sex, diabetes duration, fasting plasma glucose, hemoglobin A1c, peripheral vascular disease, diabetic retinopathy, diabetic peripheral neuropathy, hypertension, coronary atherosclerotic heart disease, chronic obstructive pulmonary disease, chronic kidney disease, stroke, osteoarthritis, cancer, and polypharmacy, metformin exposure remains negatively associated with frailty (B = − 0.572, OR = 0.564; 95% CI 0.321–0.991), independently other risk factors for frailty (Table 7).

**Table 7:** Regression models of the association of metformin and frailty

Variable	OR	95% CI	*P* value
Age	4.295	0.970–19.009	0.055
CAD	1.926	1.111–3.339	0.020
CKD	2.129	1.062–4.265	0.033
Stroke	2.875	1.560–5.298	0.001
Osteoarthritis	2.032	1.111–3.719	0.021
Cancer	3.345	1.686–6.638	0.001
PVD	2.148	1.111–4.154	0.023
Metformin	0.564	0.321–0.991	0.047
Constant	0.024		0.000

Adjusted by age, sex, duration, FPG, HbA1c, PVD, DR, DPN, hypertension, CAD, COPD, CKD, stroke, osteoarthritis, cancer, and polypharmacy. Sex, hypertension, COPD, duration, FPG, HbA1c, DR, DPN, and polypharmacy were not included in the equation

FPG, fasting plasma glucose; HbA1c, hemoglobin A1c; BMI, body mass index; PVD, peripheral vascular disease; DR, diabetic retinopathy; DPN, diabetic peripheral neuropathy; CAD, coronary atherosclerotic heart disease; COPD, chronic obstructive pulmonary disease; CKD, chronic kidney disease

### Metformin and frailty on adverse outcomes

The follow-up findings indicated that frail patients had higher rates of cardiovascular events (20.7% vs. 6.8%, *p* < 0.001), readmission (47.6% vs. 27.6%, *p* < 0.001), mortality (7.3% vs. 0.6%, *p* < 0.001), and combined primary outcomes (58.5% vs. 31.8%, *p* < 0.001) than non-frail patients. Subgroup analysis showed that metformin use was associated with a lower prevalence of cardiovascular events (1.9% vs. 15.1%, *p* < 0.001) and combined primary outcomes (26.8% vs. 40.2%, *p* = 0.010) in non-frail diabetic patients, whereas metformin use was only associated with a low prevalence of cardiovascular events (3.1% vs. 32.0%, *p* = 0.002) in frail patients with diabetes.

**Fig. 1:**
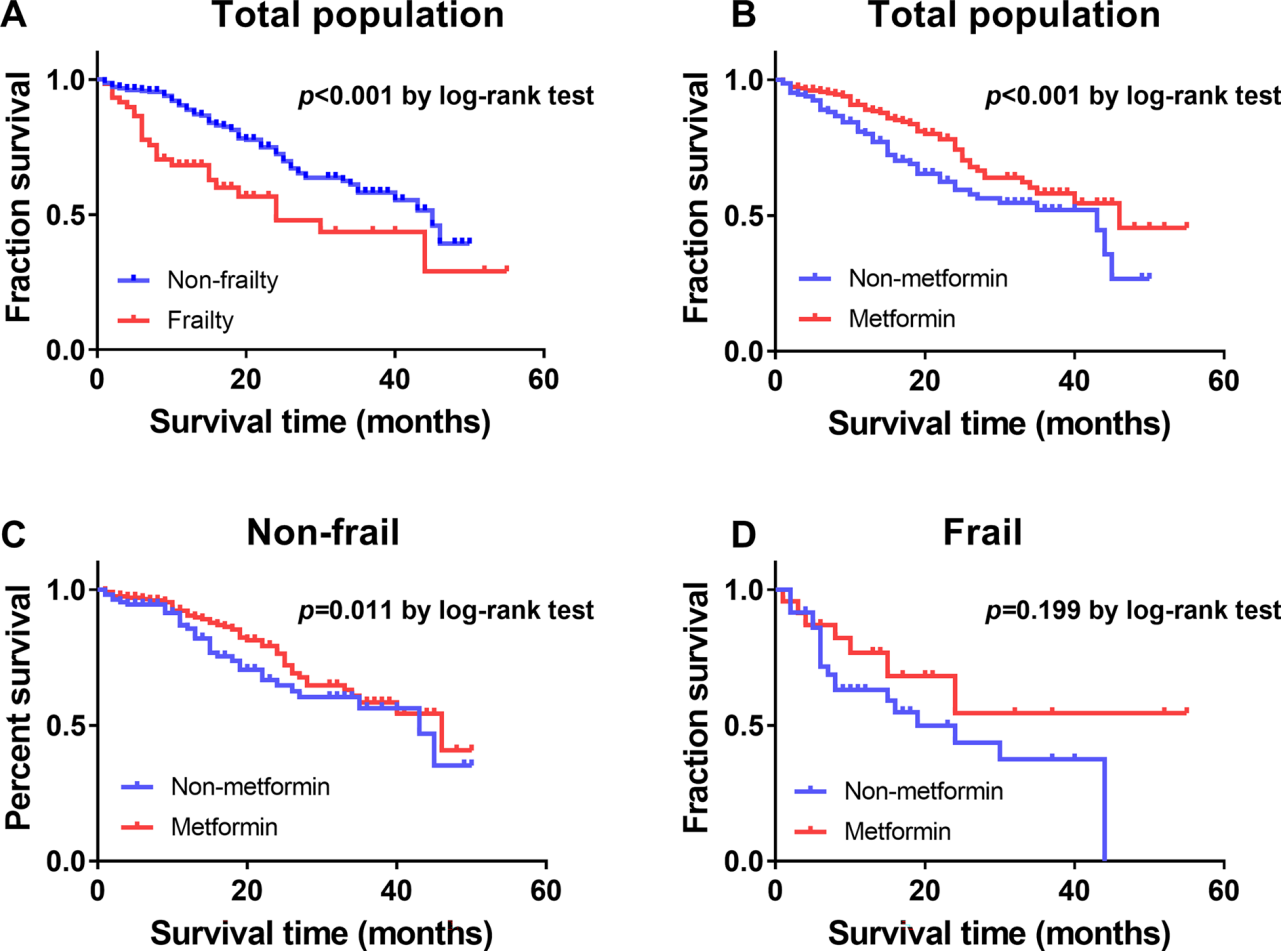
Kaplan–Meier survival curves for the combined primary outcome in months. **A** Survival time according to non-frail (*n* = 340) and frail (*n* = 82) diabetic patients in the total population. **B** Survival time according to treated with non-metformin (*n* = 177) and metformin (*n* = 245) in total population. **C** Survival time according to treated with non-metformin (*n* = 127) and metformin (*n* = 213) in non-frail diabetic patients. **D** Survival time according to treated with non-metformin (*n* = 50) and metformin (*n* = 32) in frail diabetic patients. *P* values by log-rank test

Kaplan–Meier analysis further indicated that the median survival time with and without metformin was 46.0 months (95% CI 30.52–61.48) and 24.0 months (95% CI 15.08–32.92), respectively. A significant difference in survival time was found between the non-frail and frail groups in patients with diabetes (χ2 = 30.127, *p* < 0.001; Fig. 1A). Log-rank tests indicated a statistically significant difference in survival time between the metformin and non-metformin treatment groups (χ2 = 14.266, *p* < 0.001; Fig. 1B). A similar significant difference also existed in non-frail patients with diabetes (χ2 = 6.492, *p* = 0.011; Fig. 1C) but not in frail participants (χ2 = 1.653, *p* = 0.199; Fig. 1D). In addition, Cox proportional hazard models showed that metformin treatment was independently and inversely associated with diabetes. Compared with non-metformin treatment, metformin use had a lower risk of combined primary outcome after adjusting for age and sex (hazard ratio (HR) = 0.558; 95% CI 0.407–0.765). When adjusted for frailty, duration, fasting plasma glucose and hemoglobin A1c, target organ damage, comorbidities, and polypharmacy, the protective effects remained (HR = 0.695; 95%CI 0.501–0.964). In addition, the results also indicated that frailty (HR = 2.009; 95%CI 1.408–2.865) and coronary atherosclerotic heart disease (HR = 1.699, 95%CI 1.226–2.356) were associated with the increased risk of combined primary outcomes (Table 8). In the non-frail group, metformin use was also associated with a lower risk of combined primary outcomes adjusted for age and sex (HR = 0.620; 95%CI 0.425–0.905). However, the protective effect of metformin on adverse outcomes was not found in frail participants with diabetes.

**Table 8:** Cox regression models of metformin on the primary outcomes

Variable	OR	95% CI	*P* value
Frailty	2.009	1.408–2.865	< 0.001
CAD	1.699	1.226–2.356	0.001
Metformin	0.695	0.501–0.964	0.029

Adjusted for age, sex, frailty, duration, FPG, HbA1c, PVD, DR, DPN, hypertension, CAD, COPD, CKD, stroke, osteoarthritis, cancer, and polypharmacy. Age, sex, duration, FPG, HbA1c, PVD, DR, DPN, hypertension, COPD, CKD, stroke, osteoarthritis, cancer, and polypharmacy were not included in the equation

FPG, fasting plasma glucose; HbA1c, hemoglobin A1c; PVD, peripheral vascular disease; DR, diabetic retinopathy; DPN, diabetic peripheral neuropathy; CAD, coronary atherosclerotic heart disease; COPD, chronic obstructive pulmonary disease; CKD, chronic kidney disease

## Discussion

The frailty prevalence among diabetic patients in this study was 19.4%, which was close to the 19.32% for diabetic patients in the Beijing longitudinal study of aging II [5]. Combined coronary atherosclerotic heart disease, chronic kidney disease, stroke, osteoarthritis, cancer, and peripheral vascular disease were independent risk factors for frailty in diabetic patients. Studies on older male veterans with diabetes have indicated that metformin promoted a healthy lifespan by preventing frailty and reducing the risk of age-related comorbidities and frailty-related diseases [13, 23]. In the present study, a total of 245 (58.1%) patients with diabetes were treated with metformin, 64 were on monotherapy and the other 181 participants were treated with combinations of metformin and other antidiabetic drugs. Among 177 patients not treated with metformin, 49 (27.7%) of them were treated with lifestyle interventions, 9 (5.1%) of them discontinued metformin due to gastrointestinal intolerance, and 119 (67.2%) were treated with other antidiabetic drugs because of disease conditions and metformin contraindications. The prevalence of gastrointestinal intolerance in our study was similar to the results of Genetics of Diabetes Audit and Research Tayside Study [24]. Consistent with previous findings [23, 25], we also found metformin independently and negatively associated with frailty evaluated by the Fried phenotype.

In addition, metformin use could protect against age-related musculoskeletal disorders. A study suggested that metformin treatment improved the mean walking speed in pre-frail older patients without diabetes. But they did not find any influence of metformin use on grip strength, quality of life, and myostatin serum level [14]. Other studies argued that metformin weakened the benefits of physical activity, such as resistance, endurance, and combined exercise training [15, 16]. Moreover, metformin may have no association with osteoarthritis [26]. Our study found that diabetic patients treated with metformin had better physical functions, and a lower risk of osteoarthritis than non-metformin patients. However, subgroup analyses in different frailty groups failed to determine the effects of metformin on grip strength and walking speed. Metformin was associated with a reduced risk of osteoarthritis only in non-frail patients with diabetes. Different from the opinions of Laksmi et al., our study suggested that metformin treatment improved the quality of life (assessed by the SF-36) in both frail and non-frail patients with diabetes. As the relationship between metformin use and physical function is controversial, further investigations are required [14]. MET-PREVENT is an ongoing clinical double-blind trial aiming to explore the beneficial effects of metformin on physical performance in older people with sarcopenia and physical prefrailty/frailty, which uses the 4 m walk speed at the 4-month follow-up visit as the primary outcome [27].

Metformin is a strongly recommended first-line drug for patients with type 2 diabetes, especially for those with obesity. Our findings showed that high BMI and high levels of nutrient metabolism indicators, such as blood glucose, blood lipids, and proteins, and low levels of kidney function indicators, D-dimer, and NT-proBNP were associated with metformin in diabetic patients, especially in non-frail patients. Insulin resistance and inflammation, which are common pathological processes in frailty and diabetes, are also targets of metformin. Therefore, metformin may be a promising drug for the prevention of frailty. The potential mechanisms of metformin mainly include the upregulation of nutrient-sensing pathways by activating AMP-activated kinase (AMPK), inhibiting mTORC1 [28], as well as downregulation of insulin/insulin-like growth factor-1 (IGF-1) signaling. Moreover, metformin enhances autophagy and maintains mitochondrial function to reduce age-related inflammation and reactive oxygen species [29]. A recent study found that metformin prevented female mice from age-associated ovarian fibrosis by modulating specific immune cells and fibroblasts [30].

The Targeting Aging with Metformin (TAME) clinical trial reported eight serum biomarkers associated with geoscience, including interleukin-6, CRP, tumor necrosis factor receptor II, growth differentiation factor -15, insulin, IGF-1, cystatin C, NT-proBNP, and hemoglobin A1c [31]. In the present study, high fasting plasma glucose, hemoglobin A1c, triglyceride, prealbumin, albumin, hemoglobin, urine mALB/Cre levels and low creatine, blood urea nitrogen, D-dimer, and NT-proBNP levels were associated with metformin. Low levels of CRP were only observed in frail patients with diabetes. Further comparisons found differences between serum NT-proBNP and D-dimer levels and metformin treatment. D-dimer might play a particularly important role in frailty [32, 33]. As the Cardiovascular Healthy Study mentioned earlier, high levels of D-dimer were observed in frailty older adults even after adjusting for cardiovascular disease and diabetes [33]. Those with high interleukin-6 and high D-dimer levels had the greatest functional declines [32]. Higher D-dimer and high tissue plasminogen activator was associated with an increased risk of frailty (OR = 2.20 95%CI 1.29–3.75) [34]. Cross-sectional analysis evidence indicated that elevated plasma BNP was associated with increased risks of frailty, prefrailty, and low levels of grip strength and walking speed in older adults [35]. Moreover, NT-proBNP has been identified as a simple and useful tool for frailty assessment in patients with newly diagnosed multiple myeloma [36]. High NT-proBNP levels are also strongly associated with incident frailty in the community-dwelling population without known cardiovascular disease [37]. Future studies are needed to verify the feasibility, effectiveness, predictability, and responsiveness of these serum biomarkers, as well as explore novel biomarkers.

Metformin was associated with a reduction of all-cause mortality and age-related diseases [10, 38]. Clinical studies have shown that metformin has protective effects on coronary atherosclerotic heart disease in patients with diabetes [39, 40] and coronary atherosclerosis in males with prediabetes and early diabetes [41]. A retrospective 5-year follow-up cohort study on Chinese adults with diabetes also showed that metformin treatment could reduce the risk of coronary atherosclerotic heart disease events and all-cause mortality [42]. The Glucose Lowering In Non-diabetic hyperglycemia Trial (GLINT) also found benefits of metformin for coronary atherosclerotic heart disease in non-diabetic hyperglycemia adults [43]. The follow-up results showed that metformin use in patients with diabetes was in accordance with a lower risk of adverse outcomes. Frail diabetic patients were related to high risk of adverse outcomes, including cardiovascular events, hospitalization, death, and combined primary outcomes. Consistent with the above studies, our study also found that metformin-treated patients had a significantly lower risk of cardiovascular events than non-treated patients, regardless of frailty. However, no effect of metformin on cerebrovascular disease, hospitalization, or death was observed in the subgroup analyses. Therefore, the protective effect of metformin on coronary atherosclerotic heart disease in patients with diabetes may be more sensitive than that of other adverse events. The Cox regression analysis indicated that metformin was an independent protective factor and frailty was an independent risk factor for adverse events. The protective effect of metformin on the combined primary outcomes was observed only in non-frail patients. Thus, the protective effect of metformin against adverse outcomes may be affected by the degree of frailty. One of the potential reasons is that frail diabetic patients were older and had a lower proportion of metformin treatment than that non-metformin (Table 1), which may be influenced by the disease state, the tolerance of metformin gastrointestinal adverse reactions, and the metformin contraindications. In addition, controversy exists regarding the beneficial effects of metformin on physical function. As multiple physiological changes from cellular/molecular level, system level, to organ level contribute to frailty [44]. Recently, an integrated care model is recommended for frailty management, which includes physical exercise, nutrition, comorbidity and polypharmacy management, social support, etc. [45, 46]. While research on the pharmacological treatment of frailty is still in the exploratory stage. Metformin treatment has a weaker effect on frailty, and comprehensive assessment and management are necessary for frailty management. As frailty is a dynamic process, and early frailty is reversible. The protective effect of metformin in non-frail patients may help prevent frailty progression and adverse outcomes.

There may be some possible limitations in this study. First, the sample size was small, and all participants were from a single center. There may exhibit a certain degree of selection bias. For example, frail patients treated with metformin were 13.1%, lower than that not treated with metformin (28.2%). The lower frailty proportion in the metformin group may be also influenced by disease conditions and metformin contraindications. Frailty is generally comorbid with cardiac failure, chronic kidney disease, hepatic failure and cirrhosis, and respiratory insufficiency, such as chronic obstructive diseases, which are recognized as metformin contraindications [47]. This may decrease the application of metformin in frail population. Second, comprehensive assessment and laboratory tests were not performed during the follow-up. Although we found a negative association between metformin and frailty, the clinical value was inferior to the findings from randomized controlled trials. Future research requires prospective clinical studies and validation in larger populations.

## Conclusion

In conclusion, metformin use is independently associated with a low risk of frailty in patients with diabetes. Metformin had an independent protective effect on adverse events, whereas frailty was an independent risk factor for worse outcomes. The benefits of metformin use were observed in non-frail patients with diabetes in this study, but not in those with frailty. Thus, frailty may weaken the long-term protective effects of metformin. Early identification and timely intervention of the frail state of patients with diabetes may help enhance the benefits of metformin. However, further prospective clinical studies are needed to verify these findings.

## Supplementary Information

**Additional file 1: Table S1.** Univariate regression analysis of risk factors for frailty.

## Data Availability

All data published here are under the consent for publication.

## Abbreviations

BMI: Body mass index
CRP: C-reactive protein
NT-proBNP: N-terminal pro-B-type natriuretic peptide
micALB/Cre: Microalbuminuria/creatinine
AMPK: AMP-activated kinase
IGF-1: Insulin/insulin-like growth factor-1
TAME: Targeting Aging with Metformin
GLINT: Glucose lowering in non-diabetic hyperglycemia trial
