# Supplementary material for: Association of metformin exposure with low risks of frailty and adverse outcomes in patients with diabetes
Source: Eur J Med Res. 2023 Feb 3;28:65. doi: 10.1186/s40001-023-01017-6 (PMC9896807; doi:10.1186/s40001-023-01017-6)
Supplement: Supplementary file 1 — Additional file 1: Table S1. Univariate regression analysis of risk factors for frailty. [file 40001_2023_1017_MOESM1_ESM.doc]

**Additional file: Table S1. Univariate regression analysis of risk factors for frailty**

| Variable | B | S.E. | Wald | df | P value | Exp (B) | 95%C.I. for Exp (B) | |
| --- | --- | --- | --- | --- | --- | --- | --- | --- |
|  |  |  |  |  |  |  | Lower | Upper |
| Age | 0.143 | 0.017 | 73.215 | 1 | <0.001 | 1.154 | 1.117 | 1.193 |
| Sex | 0.102 | 0.259 | 0.155 | 1 | 0.694 | 1.107 | 0.667 | 1.838 |
| Smoking | -0.104 | 0.248 | 0.175 | 1 | 0.675 | 0.901 | 0.555 | 1.465 |
| Drinking | -0.450 | 0.271 | 2.768 | 1 | 0.096 | 0.638 | 0.375 | 1.083 |
| WHR | 0.640 | 0.657 | 0.948 | 1 | 0.330 | 1.896 | 0.523 | 6.877 |
| BMI | -0.053 | 0.038 | 2.009 | 1 | 0.156 | 0.948 | 0.881 | 1.021 |
| Duration | 0.016 | 0.014 | 1.373 | 1 | 0.241 | 1.016 | 0.989 | 1.044 |
| Antidiabetic treatments |  |  |  |  |  |  |  |  |
| Lifestyle management | 0.379 | 0.337 | 1.268 | 1 | 0.260 | 1.461 | 0.755 | 2.826 |
| Metformin | -0.963 | 0.253 | 14.543 | 1 | <0.001 | 0.382 | 0.233 | 0.626 |
| Sulfonylureas | -0.040 | 0.326 | 0.015 | 1 | 0.902 | 0.961 | 0.507 | 1.821 |
| Glinide | 0.405 | 0.492 | 0.680 | 1 | 0.410 | 1.500 | 0.572 | 3.932 |
| Glucosidase inhibitor | 0.308 | 0.247 | 1.555 | 1 | 0.212 | 1.361 | 0.839 | 2.207 |
| Thiazolidinedione | 0.496 | 0.435 | 1.300 | 1 | 0.254 | 1.642 | 0.700 | 3.853 |
| DPP-4 inhibitors | -0.820 | 0.621 | 1.743 | 1 | 0.187 | 0.440 | 0.130 | 1.488 |
| SGLT2 inhibitors | -0.143 | 0.564 | 0.065 | 1 | 0.799 | 0.866 | 0.287 | 2.619 |
| GLP-1 agonist | - | - | - | - | - | - | - | - |
| Insulin | -0.191 | 0.280 | 0.466 | 1 | 0.495 | 0.826 | 0.478 | 1.429 |
| Other medicines | 0.327 | 1.161 | 0.079 | 1 | 0.778 | 1.387 | 0.142 | 13.506 |
| Comorbidity |  |  |  |  |  |  |  |  |
| Hypertension | 1.110 | 0.393 | 7.988 | 1 | 0.005 | 0.329 | 0.153 | 0.712 |
| CAD | 1.170 | 0.254 | 21.199 | 1 | <0.001 | 0.310 | 0.188 | 0.511 |
| COPD | 0.491 | 0.541 | 0.822 | 1 | 0.365 | 0.612 | 0.212 | 1.769 |
| CKD | 1.194 | 0.308 | 15.036 | 1 | <0.001 | 0.303 | 0.166 | 0.554 |
| Stoke | 1.031 | 0.271 | 14.503 | 1 | <0.001 | 0.357 | 0.210 | 0.606 |
| Osteoarthritis | 1.109 | 0.276 | 16.163 | 1 | <0.001 | 0.330 | 0.192 | 0.567 |
| Cancer | 1.322 | 0.308 | 18.420 | 1 | <0.001 | 0.267 | 0.146 | 0.488 |
| Target organ damage |  |  |  |  |  |  |  |  |
| PVD | 0.990 | 0.295 | 11.270 | 1 | 0.001 | 2.693 | 1.510 | 4.801 |
| DR | -0.491 | 0.426 | 1.327 | 1 | 0.249 | 0.612 | 0.265 | 1.411 |
| DPN | 0.707 | 0.308 | 5.290 | 1 | 0.021 | 2.029 | 1.110 | 3.707 |
| Polypharmacy | 1.187 | 0.314 | 14.330 | 1 | <0.001 | 3.278 | 1.773 | 6.061 |
| SF-36 score | -0.133 | 0.015 | 74.339 | 1 | <0.001 | 0.876 | 0.850 | 0.902 |
| Laboratory tests |  |  |  |  |  |  |  |  |
| FPG | -0.004 | 0.048 | 0.007 | 1 | 0.936 | 0.996 | 0.906 | 1.095 |
| HbA1c | -0.157 | 0.093 | 2.831 | 1 | 0.092 | 0.855 | 0.712 | 1.026 |
| Triglyceride | -0.224 | 0.137 | 2.664 | 1 | 0.103 | 0.800 | 0.611 | 1.046 |
| Total cholesterol | -0.172 | 0.115 | 2.250 | 1 | 0.134 | 0.842 | 0.673 | 1.054 |
| HDL | -0.563 | 0.422 | 1.780 | 1 | 0.182 | 0.569 | 0.249 | 1.302 |
| LDL | -0.118 | 0.139 | 0.718 | 1 | 0.397 | 0.889 | 0.677 | 1.167 |
| Creatinine | 0.011 | 0.004 | 10.179 | 1 | 0.001 | 1.011 | 1.004 | 1.018 |
| BUN | 0.270 | 0.048 | 32.203 | 1 | <0.001 | 1.310 | 1.194 | 1.439 |
| Prealbumin | -0.007 | 0.002 | 8.234 | 1 | 0.004 | 0.993 | 0.989 | 0.998 |
| Albumin | -0.195 | 0.037 | 27.778 | 1 | <0.001 | 0.823 | 0.765 | 0.885 |
| Hemoglobin | -0.037 | 0.008 | 24.150 | 1 | <0.001 | 0.964 | 0.950 | 0.978 |
| CRP | 0.004 | 0.021 | 0.039 | 1 | 0.844 | 1.004 | 0.964 | 1.047 |
| D-Dimer | 0.681 | 0.196 | 12.056 | 1 | 0.001 | 1.977 | 1.346 | 2.904 |
| Fibrinogen | 0.434 | 0.152 | 8.171 | 1 | 0.004 | 1.543 | 1.146 | 2.078 |
| NT-proBNP | 0.001 | 0.000 | 16.854 | 1 | <0.001 | 1.001 | 1.000 | 1.001 |
| Urine mALB/Cre | 0.001 | 0.001 | 3.849 | 1 | 0.050 | 1.001 | 1.000 | 1.002 |

*Abbreviations: WHR, Waist-to-Hip Ratio; BMI, Body Mass Index; CAD, Coronary Atherosclerotic Heart Disease; COPD, Chronic Obstructive Pulmonary Disease; CKD, Chronic Kidney Disease; PVD, Peripheral Vascular Disease; DR, Diabetic Retinopathy; DPN, Diabetic Peripheral Neuropathy; FPG, Fasting Plasma Glucose; HbA1c, Hemoglobin A1C; HDL, High-Density Lipoprotein; LDL, Low-Density Lipoprotein; BUN, Blood Urea Nitrogen; CRP, C-Reactive Protein; NT-proBNP, N-terminal pro-B-type Natriuretic Peptide; mALB/Cre, microalbuminuria/creatinine.*
